# Hybrid fuzzy logic–PI control with metaheuristic optimization for enhanced performance of high-penetration grid-connected PV systems

**DOI:** 10.1038/s41598-025-09336-w

**Published:** 2025-07-09

**Authors:** Mohamed Ahmed Ebrahim Mohamed, Sayed A. Ward, Mohamed F. El-Gohary, M. A. Mohamed

**Affiliations:** 1 Electrical Engineering Department, Faculty of Engineering at Shoubra, Benha University, Cairo, Egypt; 2 Faculty of Engineering, Delta University for Science and Technology, Gamasa, 35712 Egypt; 3 Projects Implementation Department, New and Renewable Energy Authority (NREA), Cairo, Egypt; 4 Solar Energy Department, National Research Centre, Cairo, Egypt

**Keywords:** Central inverters, Fuzzy logic control, High penetration photovoltaic, Optimization techniques, Power quality, Proportional integral controller, Renewable energy systems, Total harmonic distortion, Utility grid, Voltage stability, Engineering, Electrical and electronic engineering, Energy infrastructure

## Abstract

This paper introduces a hybrid fuzzy logic control-based proportional-integral (FLC-PI) control strategy designed to enhance voltage stability, power quality, and overall performance of central inverters in photovoltaic power plants (PVPPs). The study is based on a real-world PVPP with an installed capacity of 26.136 MWp, connected to the Egyptian national grid at Fares City, Kom Ombo Centre, Aswan Governorate. A user-friendly MATLAB/SIMULINK environment is developed, incorporating eleven distinct blocks along with a modelled national utility grid, utilizing actual operational data from the PVPP. To optimize the FLC-PI control scheme, several artificial intelligence (AI)-based metaheuristic optimization techniques (MOTs) are employed to simultaneously tune all control parameters—namely Grey Wolf Optimization (GWO), Harris Hawks Optimization (HHO), and the Arithmetic Optimization Algorithm (AOA)—are employed. These techniques are used to simultaneously fine-tune all the gain parameters of FLC-PI control, based on four standard error-based objective functions: Integral Absolute Error (IAE), Integral Square Error (ISE), Integral Time Absolute Error (ITAE), and Integral Time Square Error (ITSE). The optimized gains are applied to both voltage and current regulators of the central inverters, enabling the identification of optimal values. Among the tested methods, the HHO algorithm combined with the ISE objective function delivered the best performance, achieving a total harmonic distortion (THD) of 3.88%—well below the IEEE 519–2014 limit of 5.00%. The results confirm that the proposed FLC-PI controller significantly enhances the integration of high-penetration PVPPs into the utility grid by reducing power losses and inverter-induced harmonics, especially during maximum power point tracking (MPPT). Moreover, employing MOTs for controller tuning proves to be an effective solution for adapting to dynamic solar irradiance conditions. Ultimately, the optimized FLC-PI control approach enhances voltage stability, improves power quality, and boosts the overall efficiency of grid-connected PV systems.

## Introduction

Renewable energy systems (RESs) offer a promising solution to mitigate the environmental challenges associated with electricity generation from fossil fuels. However, integrating RESs with the power grid introduces additional challenges, including concerns related to reliability, harmonics, oscillations, power quality, reactive power compensation, and overall power system stability^1^. In modern power systems, the high penetration of RESs often takes priority during periods of high generation. Consequently, it is essential to satisfy a significant portion of electricity demand through RESs when available. Accordingly, the output of conventional power plants must be reduced during high-RES generation and increased when RES output is insufficient or disconnected^2^. Numerous studies have addressed the flexibility of power systems in accommodating variable RESs. Some focus on the role of RESs in supplying or consuming active power and their function as reactive power controllers, power flow controllers, and providers of inertia or short-circuit power. Other studies emphasize challenges in power transmission and distribution, including the need for harmonic filters, current limiting devices, and voltage regulation solutions^3^. RESs encompass various sources such as solar, wind, biomass, geothermal energy, and renewable hydrogen fuel cells. Solar energy is harnessed through two main technologies: photovoltaic (PV) and concentrated solar power (CSP), both relying on solar irradiation for electricity generation. Key challenges associated with solar energy at the grid interface include voltage irregularities (dips, rises, sags, swells, and pulses), frequency imbalances (over- or under-frequency), current harmonics, flicker, power interruptions, and energy losses^4^. As RESs typically use power electronic inverters or converters for grid integration, it is crucial to adhere to grid connection codes, implement accurate forecasting, enable time scheduling, incorporate energy storage systems, and ensure voltage quality compliance^5^. The integration of RESs into smart grids further necessitates precise forecasting models, reliable data for control and operation, and effective trading strategies. Moreover, techniques such as fault detection, network design, and power flow optimization play a vital role in enhancing grid stability and the economic efficiency of smart grid operations^6^. The high penetration of photovoltaic (PV) systems into distribution networks introduces several challenges to the functionality of power systems, particularly affecting stability, protection, operational management, and planning. These challenges primarily stem from the nonlinear behavior of PV systems. As a result, it is essential to redesign and adapt control, management, and protection strategies to ensure reliable grid operation^7^. Among various solar technologies, on-grid PV systems play a significant role in supplying electricity to the utility grid. In these systems, the generated direct current (DC) power is regulated using DC-DC boost or buck converters and subsequently converted into alternating current (AC) by inverters, in compliance with grid code standards. However, as the penetration level of PV systems continues to rise, their intermittent generation introduces adverse effects such as reverse power flow, complications in protection coordination, increased feeder losses, reduced supply security, power quality issues, and voltage oscillations^8^. These problems are further influenced by local weather variations and the characteristics of power electronic devices associated with PV inverters at the point of grid interconnection^9^. To address these issues, it is necessary to enhance the operation of voltage regulators (VRs) through increased switching actions in Volt-Var/Watt control modes, reactive power regulation, and power factor adjustment^10^. Additionally, the functionality of VRs and on-load tap changers in transformers is significantly impacted by the high integration of grid-connected PV systems. Therefore, establishing effective coordination between PV installations and voltage control devices is critical to mitigate voltage rise and flicker across the grid. Research indicates that when PV integration remains below 15% of the system’s peak load, its impact on the grid is negligible. However, exceeding this threshold can compromise the system’s stability and reliability^11^. In modern power systems, the integration of smart grid technologies is crucial for secure and efficient operation. Smart grids facilitate reliable data exchange and enable the application of optimization algorithms and computational intelligence techniques to enhance the performance of distribution control centers^12^. To mitigate the adverse effects of increasing PV penetration, various strategies such as voltage regulation, coordinated control mechanisms, and the deployment of smart inverter functionalities are essential^13^. A power management strategy that integrates a proportional-resonant current controller, DC voltage regulator, maximum power point tracking (MPPT) controller, AC voltage synchronization panel, high PV penetration detector, active power controller, and energy storage units has been proposed to enhance power quality and improve power flow in grid-connected PV systems^14^. Several studies have shown that in medium-voltage networks, overhead cables are more capable of accommodating PV plants compared to underground cables. Additionally, the power system’s ability to integrate PV plants improves with higher system frequencies^15^. However, the high penetration of grid-connected PV plants poses significant challenges to medium-voltage networks due to the nonlinear and unpredictable nature of their generation. Moreover, factors such as improper site selection, incorrect plant sizing, weather conditions, and fluctuating load demands can negatively affect the performance of distribution networks^16^. As the integration of grid-connected PV plants increases, there is also a corresponding rise in the demand for reactive power, which PV systems themselves cannot fully supply. This leads to a reduction in the total harmonic distortion (THD) of the grid voltage, while the total demand distortion (TDD) of the grid current increases. To mitigate the effects of THD and TDD—particularly at the 3^rd^, 5^th^, and 7^th^ harmonics, which are generated by high PV penetration, it is recommended to use filters in the central inverters of PV plants^17^. Egypt, situated within the solar belt, is particularly rich in solar energy. The country experiences an average annual solar irradiation of 2000–3200 kWh/m^2^, with daily sunlight hours ranging from 9 to 11 h, offering a solar energy potential of 74 billion MWh per year. Currently, PV systems contribute over 2% of Egypt’s total electricity generation, making solar energy the second-largest renewable source. According to the Egyptian government’s strategy, this share is expected to reach 22% by 2035^18,19^. Despite this promising potential, Egypt’s energy sector faces challenges due to the intermittent and nonlinear generation characteristics of renewable energy sources (RESs), which manifest in issues such as inertia shortages, frequency fluctuations, low power quality, harmonics, resonance, and voltage instability^20^. The disconnection of a significant portion of grid-connected PV plants during voltage sags leads to a dramatic drop in voltage stability. Therefore, it is recommended to continue the integration of these plants, particularly during voltage disturbances, as failing to do so may hinder voltage recovery during and after faults. To address the nonlinearity of grid-connected PV power plants during electrical peak overloads, advanced management systems are essential for defining available energy sources and demand. This allows for improved control over the utility grid, enhances power system stability, and prevents power failures^21^. THD levels in the power system are influenced by factors such as the share of PV plants in the distribution network, the location of PV plants relative to the grid, harmonic resonance effects, and the fluctuating generation of PV inverters due to variations in solar irradiance^22^. Additionally, the large-scale integration of PV power plants, especially when coupled with irregular loads and reverse power flows in distribution networks, exacerbates THD and affects grid voltage levels^23^. However, integrating high-penetration PV plants closer to load centers can reduce power losses, particularly under heavy inductive loads, improve the voltage profile, and prevent voltage collapse. This comes at the cost of a slight increase in short-circuit current levels in the utility grid^24^. Moreover, the non-unity power factor caused by inverters in high-penetration PV systems can lead to reduced overall power factor in distribution networks, resulting in insufficient active power, inadequate reactive power supply, overheating of electrical equipment, increased voltage drops, and switching failures. Reactive power compensation is an effective method for optimizing voltage and frequency stability in the utility grid during periods of high PV penetration. However, as reactive power compensation increases, so does the risk of instability in frequency control^25,26^. High penetration of distributed PV plants into the utility grid causes significant impacts on power quality, including decreased reliability, shorter equipment lifespans, increased total harmonic distortion (THD), higher power losses, voltage fluctuations (both overvoltage and undervoltage), harmful effects on sensitive equipment, reverse power flow, protection system malfunctions, and deviations from the nominal system frequency. To address these challenges, it is recommended to implement several changes in the power system, such as upgrading protection systems to handle bidirectional power flow, incorporating islanding protection, and resizing network components (e.g., grounding, short-circuit protection, breaking capacity) along with supervisory control and data acquisition (SCADA) systems to improve power quality^27^. Furthermore, the connection point between the PV power plants and the grid should be equipped with dynamic voltage regulators to manage voltage sags, flicker, imbalance, frequency variations, and power factor. An RLC filter is also recommended to reduce current and voltage harmonics^28^. The high penetration level of distributed PV generation must be within the maximum capacity limits of the network. Exceeding this capacity can lead to excessive harmonic distortions, affecting overall power system performance and exacerbating power quality issues within the distribution network^29^. On the other hand, integrating distributed PV generation into the grid can enhance the loading margin and improve the voltage magnitude of the power system^30,31^. To mitigate power quality challenges in weak networks with high PV penetration, various devices from distributed flexible AC transmission systems (DFACTS) and control methods (including conventional, adaptive, and AI-based optimization algorithms) are essential^32^. For instance, a combination of optimization algorithms such as Binary Particle Swarm Optimization (BPSO) and Grey Wolf Optimization (GWO) can be used to enhance the nonlinear generation of PV power plants by controlling voltage and power characteristics through several MPPT methods (such as hill climbing, perturb and observe, and incremental conductance). This helps reduce THD during variations in temperature and solar irradiation^33^. Additionally, integrating electrical filters such as passive filters, shunt active power filters, and hybrid active–passive filters with the solar inverters of PV power plants can significantly reduce THD and improve overall power quality. Shunt hybrid filters have proven to be effective in enhancing performance and power quality for PV plants connected to the smart grid. It is also recommended to apply advanced control techniques like adaptive fuzzy neural networks to achieve efficient operation of the smart grid under various load and supply voltage conditions^34^. For reactive power compensation, improving the voltage profile, reducing losses, and increasing the power transfer capability of grid-connected hybrid renewable energy systems, the use of a static VAR compensator (SVC) is recommended^35,36^. The high penetration of PV plants can create harmful impacts on the utility grid, such as reverse power flow, overvoltage, and undervoltage, leading to repeated disconnections of solar inverters when voltage exceeds the normal maximum level^37^. Increasing PV penetration in the utility grid can also cause overload conditions in substations due to reverse power flow, which in turn can reduce the lifetime of transformers. The Optimal Power Flow (OPF) is crucial for optimizing control variables (e.g., active power generation, bus voltages, transformer tap ratios, and reactive power of shunt compensators) and minimizing constraints in the operation and planning of electric power systems that incorporate renewable energy sources. Optimization algorithms can be employed to solve OPF problems, such as total fuel cost, emissions, power losses, and voltage magnitude deviations, especially when integrating traditional thermal power plants with intermittent renewable energy sources^38^. Several artificial intelligence (AI) algorithms, such as Particle Swarm Optimization (PSO), Moth-Flame Optimization (MFO), Grey Wolf Optimization (GWO), Ant Colony Optimization (ACO), Sine Cosine Algorithm (SCA), and Harris Hawks Optimization (HHO), can be used to optimize these OPF problems^39,40^. This paper presents a simulation of a 26.136 MWp grid-connected PV power plant, already integrated into the Egyptian electricity grid at Fares City, Kom Ombo Center, Aswan Governorate, since 2020. The authors have constructed user-friendly MATLAB/SIMULINK models to replicate the existing PV power plant using actual site data. Additionally, a control method combining Fuzzy Logic Control (FLC) and Proportional-Integral (PI) controllers is developed to improve the performance of the central inverters under varying solar irradiation conditions. Optimization techniques are employed to tune the gains of the proposed FLC-PI controller. The 26.136 MWp PV power plant consists of eleven blocks, each containing 7200 PV modules with a capacity of 330 Wp per module, arranged in arrays to achieve a total capacity of 2376 kWp per block. The DC power generated by each block is connected to DC-DC boost converters, which use the Perturb & Observe (P&O) method for Maximum Power Point Tracking (MPPT). These DC-DC boost converters are part of the central solar inverters, which convert the generated DC power to AC power in accordance with grid code regulations. To mitigate the negative impacts on power system performance and reduce the higher total harmonic distortion (THD) at the grid caused by the high penetration of PV power plants, meta-heuristic optimization techniques (MOTs) are employed. These MOTs can optimize the gains of the proposed Fuzzy Logic Control (FLC)-PI controllers for both the voltage and current regulators of the central inverter under varying sunlight conditions. In this paper, the Grey Wolf Optimization (GWO), Harris Hawks Optimization (HHO), and Arithmetic Optimization Algorithm (AOA) are applied as MOTs to optimize the gains of the FLC-PI control for this PV power plant. The result is a significant enhancement in voltage stability and power quality. Additionally, incorporating LC filters into the PV power plant can effectively limit voltage transients, power fluctuations, and THD, particularly under partial shading conditions.

### The research gaps

Despite the significant advancements in integrating high-penetration PV power plants into utility grids, several challenges remain unresolved. Most existing studies either focus on small-scale PV systems or lack detailed simulation models that reflect real-world utility-scale PV plants. Moreover, the nonlinear and uncertain nature of PV generation under varying sunlight conditions, especially partial shading, leads to poor voltage stability and high total harmonic distortion (THD), which are not adequately mitigated by conventional control strategies. While fuzzy logic and PI controllers have been explored individually, limited research combines them into a hybrid FLC-PI structure with optimized performance using modern meta-heuristic optimization techniques. Additionally, few works have validated such strategies on large-scale grid-connected PV plants using real operational data, particularly in regions like Egypt where solar potential is high but grid infrastructure faces stability and quality issues.

The major contributions of this paper can be summarized as follows:

- A user-friendly MATLAB/SIMULINK model for a realistic 26.136 MWp large-scale grid-connected PV power plant, already integrated into the Egyptian electrical network in Fares City, Kom Ombo Center, Aswan Governorate, Egypt has been constructed.
- A control method combining FLC with PI control to enhance the performance of the central inverters in this PV power plant has been developed.
- Several modern optimization techniques, including GWO, HHO, and AOA, have been applied to optimize the gains of the FLC-PI control, thereby improving power quality and voltage stability under different sunlight conditions and partial shading scenarios.
- An in-depth analysis has been conducted to pave the way to provide solutions for the integration of high-penetration PV power plants into the Egyptian network, ensuring high performance, greater efficiency, and lower THD

The remainder of this paper is organized as follows: In Sect. **“**Optimization algorithms”, the proposed optimization framework, encompassing mathematical models for GWO, HHO, and AOA is developed. This section also provides the pseudocodes as well as flow charts for the proposed optimization algorithms. Section “Photovoltaic power plant simulation” illustrates the construction of PV power plant. The application of the proposed FLC-PI tuned using the suggested optimization methods for the constructed PV power plant, is demonstrated in Sect. “Simulation and results analysis”. Moreover, the results and discussion are presented also in this section. Finally, Sect. “conclusion” presents the conclusion.

## Optimization algorithms

**Fig. 1:**
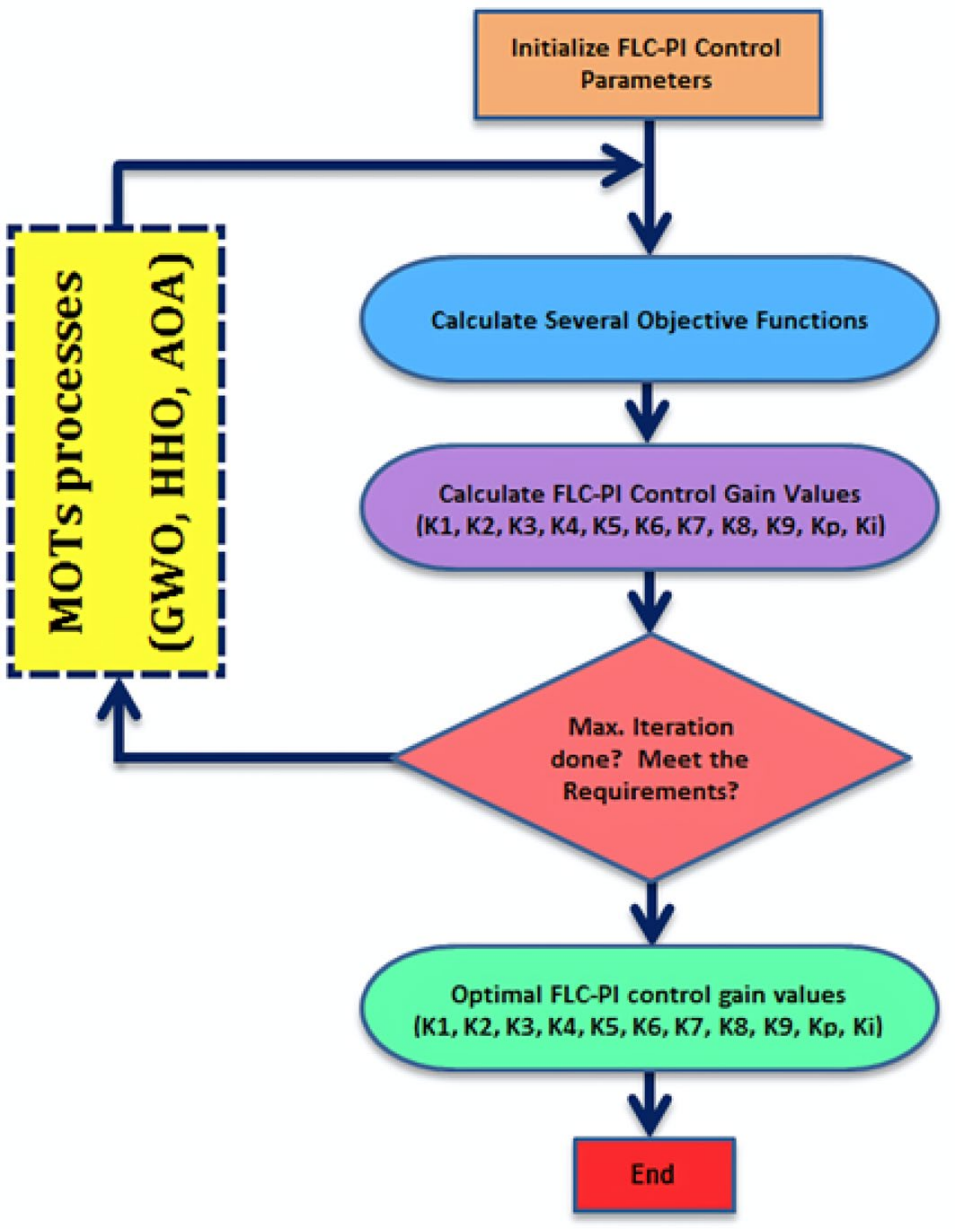
Flowchart of the optimal FLC-PI control gains generated using MOTs.

In this paper, optimization techniques such as Grey Wolf Optimization (GWO), Harris Hawks Optimization (HHO), and Arithmetic Optimization Algorithm (AOA) are artificial intelligence algorithms used for meta-heuristic optimization techniques (MOTs). These MOTs optimize all the gains of the Fuzzy Logic Control (FLC)-PI controller for both the voltage and current regulators of the central inverters in the proposed PV power plant under different sunlight conditions. The gains to be optimized for the FLC-PI control include the proportional gain (Kp) and integral gain (Ki) for the PI controller, as well as K1, K2, K3, K4, K5, K6, K7, K8, and K9 for the FLC controller. The voltage and current regulators of the central inverters in the proposed PV power plant are adjusted by optimizing all of these gains simultaneously. As a result, the dynamic performance and system interface of the proposed grid-connected PV power plant are significantly improved. To illustrate the steps involved in generating the optimal FLC-PI control gains using MOTs, a flowchart is provided in Fig. 1.

### Grey wolf optimization

Grey Wolf Optimizer (GWO) algorithm is a meta-heuristic inspired by grey wolves (Canis lupus) which imitate the leadership hierarchy and hunting mechanism of grey wolves in nature. There are four types of grey wolves such as alpha; beta, delta, and omega are employed for simulating the leadership hierarchy. In addition, the three main steps of hunting, searching for prey, encircling prey, and attacking prey, are applied. The Pseudo-code of GWO is described in Algorithm 1 to show how GWO manner is theoretically able to solve optimization problems^41^. The Grey Wolf Optimizer (GWO) algorithm is inspired by the social hierarchy and cooperative hunting behaviour of grey wolves in nature. In this algorithm, the search process mimics the wolves’ strategy of surrounding and attacking prey, where the hierarchy is defined as α, β, γ, and δ wolves, representing the best, second-best, and third-best solutions, followed by the rest of the population. The α wolf typically guides the hunting process, while β and γ support the guidance and decision-making. During the optimization, the wolves encircle the prey (i.e., the optimal solution) and adjust their positions accordingly to simulate an effective hunt^42^.

**Algorithm 1:**
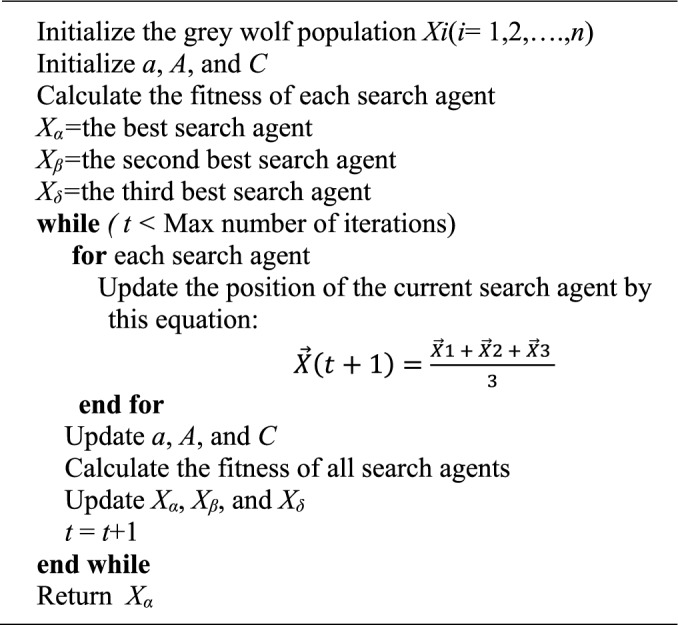
Pseudo-code of the GWO algorithm.

The GWO algorithm demonstrates strong convergence characteristics due to its balanced trade-off between exploration (searching new areas) and exploitation (refining known good areas). Its main advantages include simplicity, minimal parameter tuning, and the ability to operate without needing gradient information of the objective function. Furthermore, it is well-suited for modern optimization problems due to its flexibility and ease of implementation. However, one of the limitations of GWO is its tendency to become trapped in local optima and its relatively slow convergence speed in certain complex scenarios^43^.

### Harris hawks optimization

The Harris Hawks Optimization (HHO) algorithm is inspired by the cooperative hunting behaviour and surprise pouncing tactics of Harris’ hawks in nature. These birds exhibit a strategic and intelligent group-hunting mechanism, where multiple hawks simultaneously approach the prey from different directions to maximize the success of the attack. This dynamic and adaptive behaviour is modelled in HHO to effectively address optimization problems. The algorithm mimics various phases of hawk hunting, including exploration (foraging), exploitation (targeting), surprise pounce (rapid convergence), and diverse attacking patterns, which are determined based on the prey’s escape strategies and environmental conditions. This adaptive hunting strategy allows the HHO algorithm to balance global exploration with local exploitation efficiently. The detailed steps and behaviour of the algorithm are outlined in the pseudo-code presented in Algorithm 2, which demonstrates the algorithm’s theoretical capability in solving complex optimization tasks^44^. HHO algorithm has shown the capability to produce distinct and competitive solutions compared to many well-known metaheuristic optimizers. However, it faces limitations when applied to high-dimensional optimization problems. Its reliance on stochastic search behaviour can lead to insufficient population diversity and premature convergence, making it prone to becoming trapped in local optima^45^. Additionally, HHO exhibits certain drawbacks such as reduced accuracy, relatively slow convergence speed, and a tendency to stagnate around suboptimal solutions during the optimization process^46^.

**Algorithm 2:**
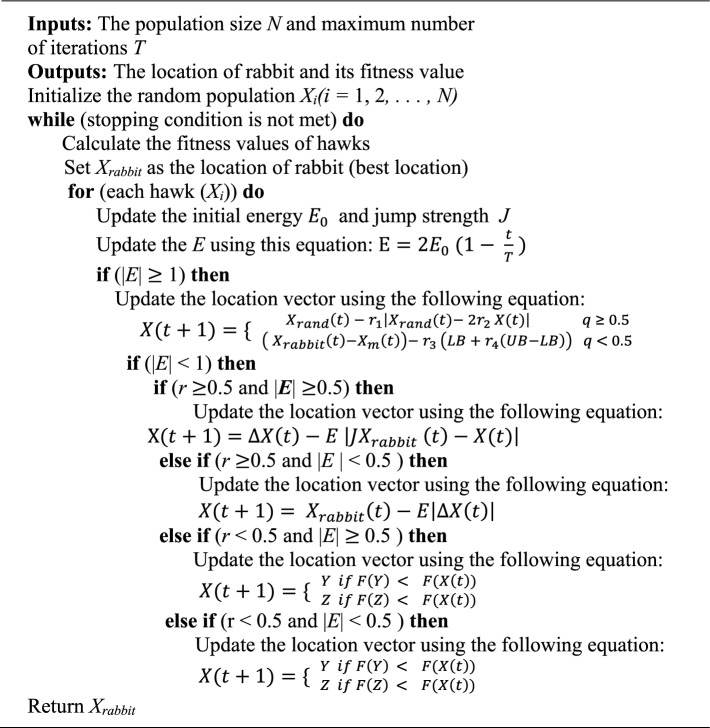
Pseudo-code of the HHO algorithm.

### Arithmetic optimization algorithm

The Arithmetic Optimization Algorithm (AOA) is a metaheuristic inspired by the fundamental principles of arithmetic operations—multiplication, division, subtraction, and addition—which are utilized to construct its mathematical framework. These operations guide the algorithm through both exploration and exploitation phases across a broad search space, enabling it to effectively navigate complex optimization problems. AOA is characterized by its fast convergence rate, efficiency, and ability to produce competitive global search results. It has demonstrated superior performance on various benchmark functions, achieving high-quality solutions in relatively shorter computation times when compared to several conventional optimization algorithms. The theoretical foundation and procedural structure of AOA are outlined in the pseudo-code provided in Algorithm 3, illustrating its capability to solve diverse optimization challenges^47^. AOA demonstrates superior computational performance compared to many competing optimization techniques. However, it still encounters several limitations. These include the risk of being trapped in local optima, challenges in accurately updating candidate solutions toward the global optimum, premature convergence, and limited solution accuracy. Moreover, AOA often struggles with maintaining sufficient exploration capabilities, particularly in high-dimensional search spaces, leading to convergence toward sub-optimal solutions in complex optimization problems^48^.

**Algorithm 3:**
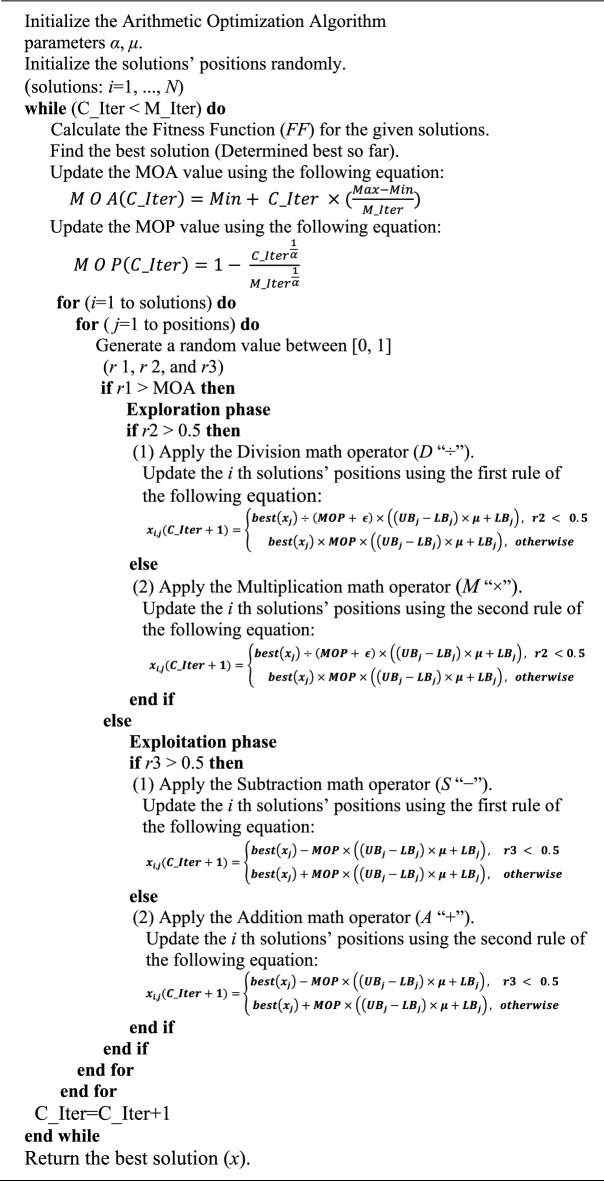
Pseudo-code of the AOA algorithm.

## Photovoltaic power plant simulation

**Fig. 2:**
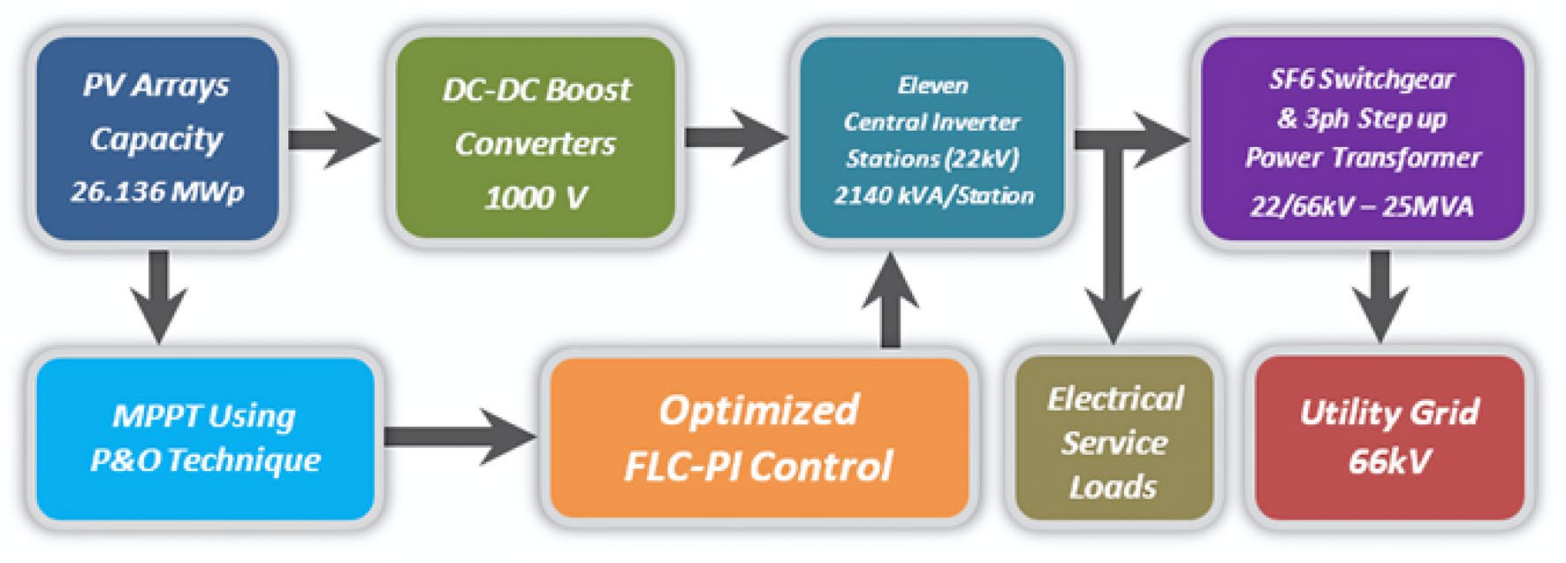
A schematic diagram for the simulated PV power plant parts.

This paper presents a simulation for 26.136 MWp grid connected PV power plant already tied to the Egyptian electricity grid at Fares city, Kom Ombo centre, Aswan governorate, Egypt since 2020. Simulation of this PV power plant is built by MATLAB/SIMULINK using actual data of the existing PV station. To show the sequence between all the simulated parts of this PV power plant, there is a schematic diagram for these parts presented in Fig. 2.

### Photovoltaic arrays

**Fig. 3:**
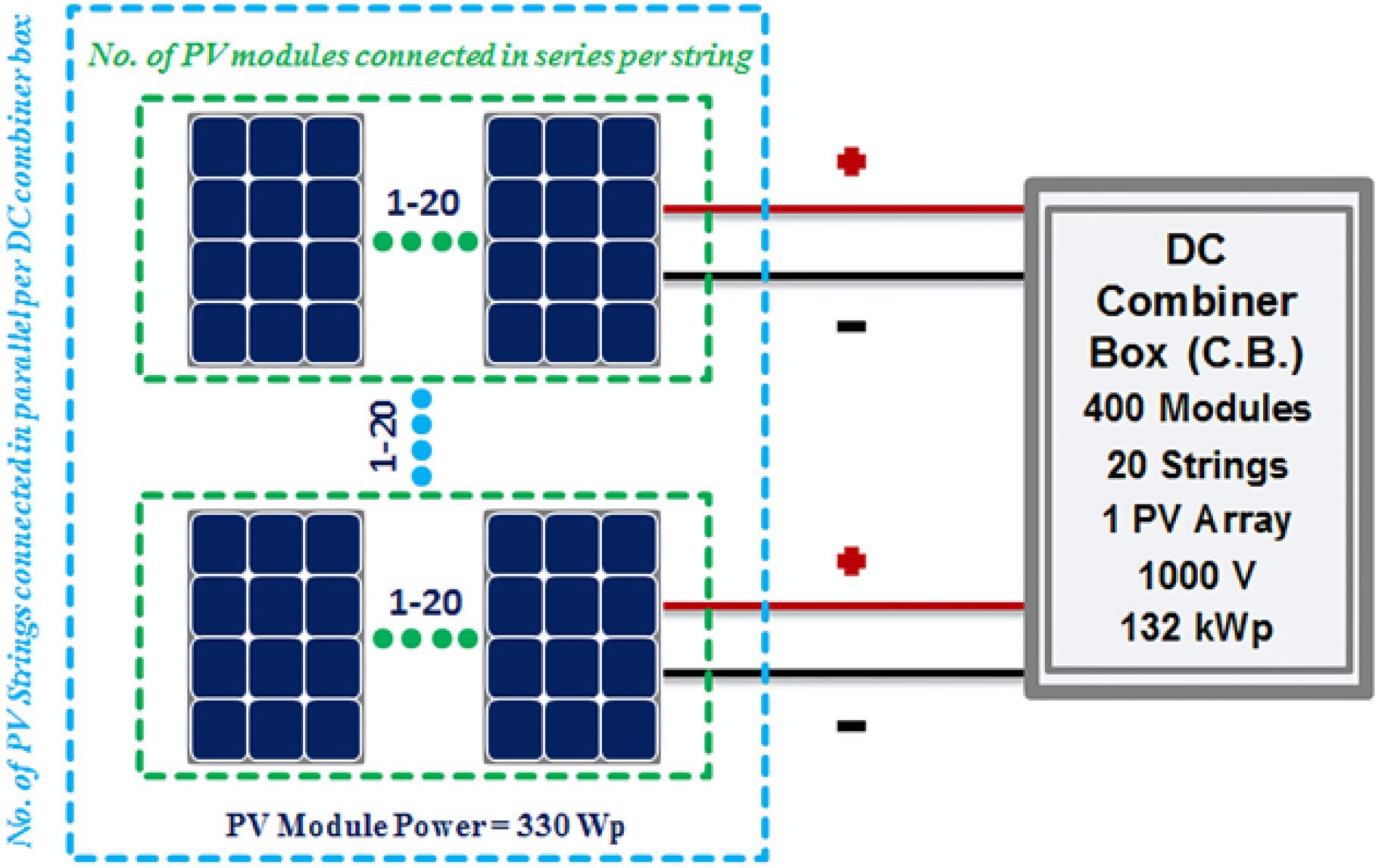
Connection sequence between photovoltaic strings and every DC combiner box.

PV arrays are DC power generators of the simulated 26.136 MWp PV power plant that consists of eleven blocks and the access point to the utility grid. All components of these eleven blocks and the access point to the utility grid are simulated by using MATLAB/SIMULINK. PV modules used to build strings of these eleven blocks are simulated by a current source coupled with other electrical elements^49^. Every block of this PV power plant contains 7200 PV modules where the power of each one PV module is 330 Wp, connected in arrays to be with total capacity 2376 kWp/block. Every array contains number of PV strings that are connected in parallel inside DC combination box as is shown in Fig. 3.

### DC-DC Boost converter and maximum power point tracking unit

**Fig. 4:**
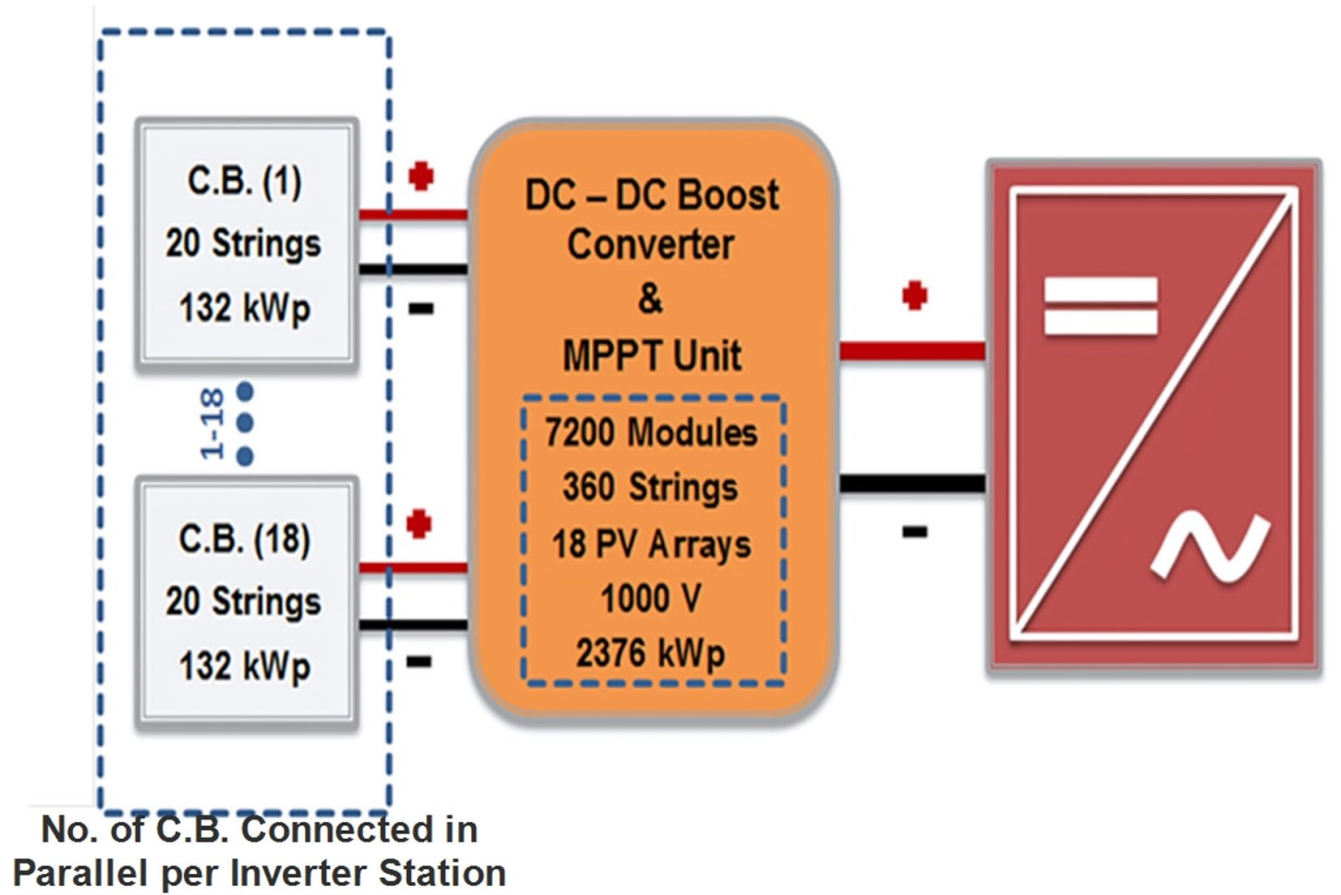
Line diagram between DC combiner boxes and every central inverter station.

The fluctuated characteristics of PV arrays lead to nonlinearity in the output I-V and P–V curves, because of sudden changes in sun irradiation level and temperature. A self-automated control method called the MPPT algorithm forces the PV system to run at maximum power point (MPP) in order to capture the most power possible given during varying environmental factors as temperature, solar irradiation, PV module characteristics, and shading^50^. Consequently, it is necessary to follow the maximum power carefully through using MPPT algorithm with DC-DC boost converter to increase the possible generated DC power of PV arrays without any oscillation^51^. The MPPT algorithm generates a PWM gating signal for the DC-DC boost converter’s power switch to regulate its output by using the PV system’s output voltage and current^52^. The Perturb and Observe (P&O) method is one of MPPT methods/algorithms that is applied here on DC-DC boost converters of the proposed PV power plant to increase its efficiency, performance, and the generated DC power during a better response time^53,54^. The simulated PV power plant contains eleven central inverter stations, and every inverter station has eighteen DC combiner boxes connected in parallel through DC boost converter integrated in MPPT unit which regulate the delivered DC power to energize the inverter as is shown in Fig. 4.

### On grid central inverter station

**Fig. 5:**
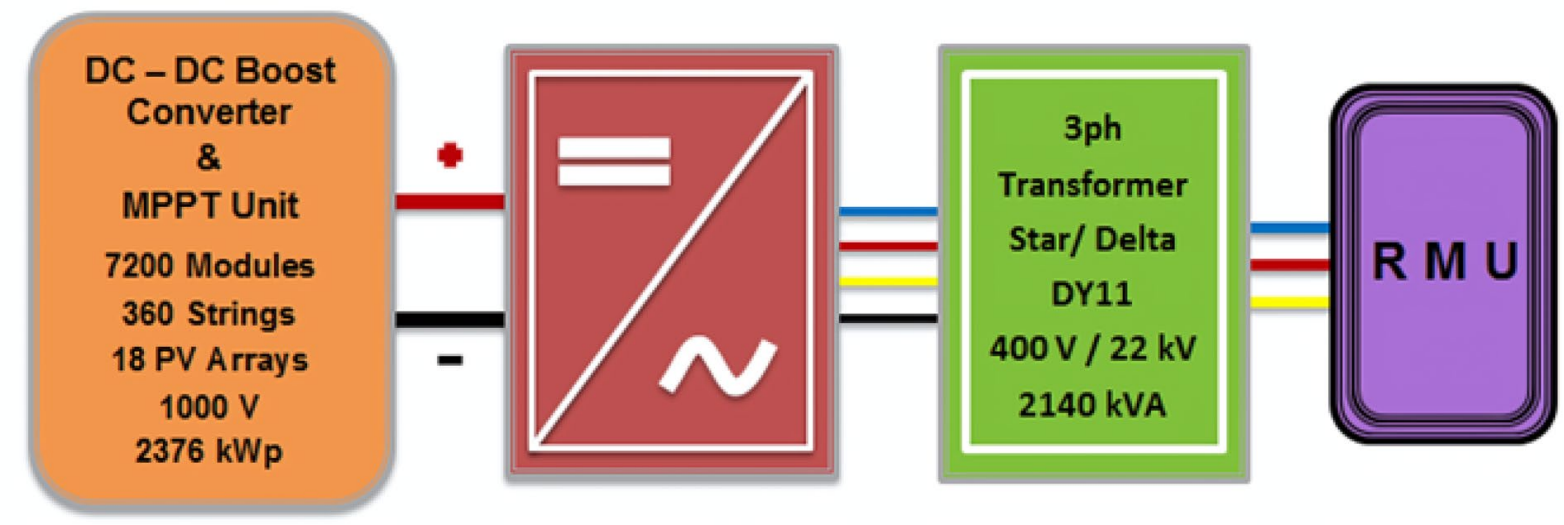
Central inverter station components connected with ring main unit (RMU).

The output DC power of PV arrays transfers through DC-DC boost converters to regulate and modify it especially during abnormal effect of different sunlight conditions or partial shading. The output DC power of these boost converters is injected in grid connected central inverters which convert it directly to AC power. Using IGBTs (Insulated Gate Bipolar Transistors) as a switching device, these central inverters perform DC-AC conversion and are connected to the grid in accordance with the Egyptian grid code’s regulations^55^. The proposed PV power plant contains eleven central inverter stations; capacity of each station is 2140 kVA, and every station has one power transformer used to step up output 3ph voltage to be 22 kV and all these stations connected together by cabling of Ring Main Unit (RMU) as is shown in Fig. 5.

### Central inverters control

This paper presents FLC-PI control based MPPT strategy for controlling central inverters of the proposed PV power plant. FLC-PI control integrates two levels of control architecture, which combines the advantages of both FLC and PI controller. FLC-PI control helps to convert PV power easily in a fast dynamic response and tracks reference current with a low overshot and short settling time especially during the variation of solar irradiance and shading effect as a result, it leads to decrease fluctuations of the power system performance^56,57^. Additionally, compared to the conventional techniques, the FLC-PI control-based MPPT strategy can achieve better efficiency, insignificant oscillations around the MPP, and an effective MPP tracking speed under different atmospheric conditions^58^. This control has a set of gains which are divided into Kp, Ki for PI controller and K1, K2, K3, K4, K5, K6, K7, K8, K9 for FLC. Adjusting voltage and current regulators of these central inverters are optimizing all these gains at the same time. Consequently, the dynamic performance, the interface system, and the quality factors of the proposed PV power plant with the utility grid can be improved. Also, FLC-PI control assures achievement of the maximum power point under different climatic conditions and provides robust responses in rapid variation of load^59^. Using FLC-PI control with pulse width modulator (PWM) and filters in the central inverters of this PV power plant led to enhance output voltage waveform, frequency spectrum, Power quality, and represses THD. Combination of PV generation with conventional thermal power plants into the utility grid requires applying power management control as the proposed FLC-PI control which can achieve the balance between generation and load alteration rapidly, hence normalizes frequency, voltage, and reactive power of the power system in shortest possible time^60,61^. Also, synchronization of the grid connected central inverters needs control system to be more accurate and efficient with variations/disturbances of the utility grid, as FLC-PI control which can mitigate the disruption of the network (jump to phase, change of amplitude, harmonics, disequilibrium…) in a fast time^62^. Integration of FLC with PI controller for interacting with the three phase central inverters increases quantity of the electric energy generated of PV power plant into the utility grid and reduces value of THDs produced in the voltage/current waveform after the filtering, more than applying FLC or PI controller separately under the same value of the solar radiation intensity^63^. Tuning all the gains of FLC-PI control by the optimization algorithms requires reducing the discrepancy between the actual and target values to achieve the objective function. In this paper, integral of absolute error (IAE), integral of square error (ISE), integral of time and absolute error (ITAE), and integral of time square error (ITSE) are the four error benchmark objective functions that are used during the optimization processes for all these gains^64^. The optimization algorithms are AI applied on the parameters of the proposed FLC-PI control to tune its value and to upgrade the overall performance of this control as a result improves the energy conversion efficiency of central inverters for the simulated PV power plant, minimizes the frequency fluctuations and limits transmission oscillation in the power system specially under different environmental conditions, high penetration level of RESs, and changes in the demand^65^. Also, using the optimized FLC-PI control can enhance the transient response and robustness characteristics of the automatic voltage regulator (AVR) inserted in the central inverters of the proposed PV power plant therefore, a faster settling time in the step responses with maximum overshoot almost equal zero is achieved^66^.

### Switchgear, transmission substation, and utility grid

**Fig. 6:**
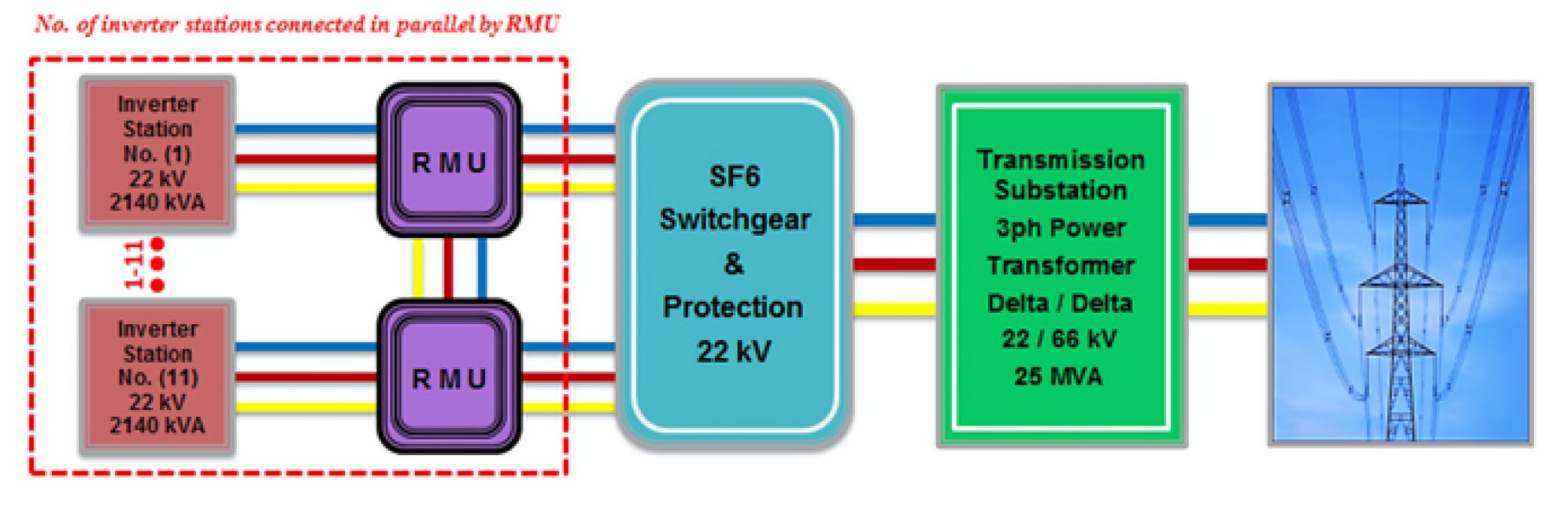
Electrical connection between the proposed PV power plant parts.

Switchgear provides suitable apportionment of the MV system to reduce the extent of circuit outages during electrical work on cables and power stations. Switchgear helps to distribute and carriage of load and overloads during safe testing, operation, maintenance and service of different electrical connectors in addition to de-energizing equipment, identify various types of faults in connection failures, and minimize the damage of the power system equipment (circuit breakers, controllers, protectors, isolators, regulators, Lightning Arresters, Relays, Fuses, Switches, current/potential transformers, and meters). There are three classes of switchgear systems according to voltage (low voltage, medium voltage, and high voltage); in the proposed PV power plant (22 kV-SF6) medium voltage switchgear is used. Utility grid is an interconnected network for electricity transportation from the power generators (Conventional and RESs) to electrical loads through an access point which is located on the network to consume or inject electricity. Utility grid consists of a set of power stations, electrical substations that are used to step voltage up or down, electric power transmission to carry power over long distances, and finally electric power distribution to customers. The proposed PV power plant contains eleven central inverter stations connected using RMU which transfer the generated AC power through SF6 medium voltage switchgear to the transmission substation by cables. According to the practical design of the proposed PV power plant, there is a power transformer (22/66 kV-25MVA) in the transmission substation. This power transformer is used to step up the produced voltage of the central inverter stations from (22 kV) to (66 kV) as the utility grid voltage. Electrical connection between the proposed PV power plant parts (inverter stations, RMU, switchgear, power transformer, and the access point with the utility grid) as shown in Fig. 6.

## Simulation and results analysis

### Simulation environment and parameter settings

**Fig. 7:**
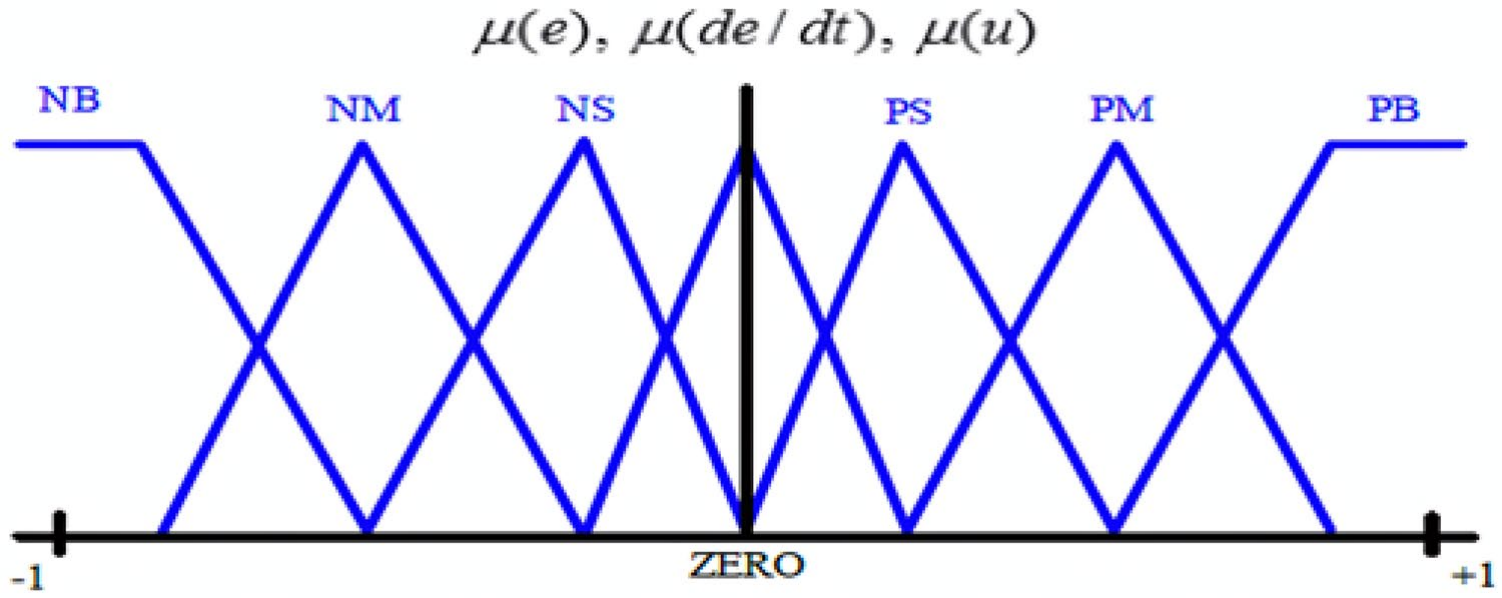
Membership functions of error (e), change of error (de/dt), change of output (u).

Simulation of the proposed PV power plant parts is built by MATLAB/SIMULINK through using actual data of the prepared technical design for establishing it. Also, this simulation presents a proposed control method which combines FLC with PI controllers to improve performance of the central inverters for this PV power plant during different solar irradiation conditions. In addition to using AI optimization techniques in steps of this simulation to tune all the gains of the proposed FLC-PI control. AI optimization techniques can optimize FLC-PI control parameters through defining the best value of these gains which are divided into Kp, Ki for PI controller and K1, K2, K3, K4, K5, K6, K7, K8, K9 for FLC at the same time. Consequently, adjusting voltage and current regulators setting are achieved then can control the three levels IGBTs Bridge of these central inverters. GWO, HHO and AOA are AI optimization techniques that are used to tune all FLC-PI control gains then optimize all parameters of the central inverter regulators as a result enhancing voltage stability, power quality, and the overall performance of this PV power plant are achieved. In the simulation of this PV power plant all the LC filters built in the central inverters are adjusted also to limit voltage transient and power fluctuation are produced into the utility grid during the partial shading effect. In general, every optimization method requires objective function for reducing the discrepancy between the actual and target values during tuning all the gains of FLC-PI control by the optimization algorithms. IAE, ISE, ITAE, and ITSE are the four error benchmark objective functions that are used with the process of optimization methods (GWO, HHO and AOA) for tuning all the gain values of the proposed FLC-PI control. Also, there are (49) rules of input variable for FLC (error, change of error) that must be generated from the seven membership functions, to design the fuzzy membership functions and fuzzy rules as shown in Fig. 7 and Table 1^67,68^. These rules help in making decisions or control actions when the system deals with nonlinear, uncertain, imprecise, or vague data. Fuzzy rules are typically constructed based on expert knowledge, observations, or data. Fuzzy rules can be constructed according to the following steps: (i) Defining inputs/outputs; (ii) Creating linguistic variables and membership functions; (iii) Using expert knowledge or data, (iv) Writing IF–THEN rules; (v) Testing and refining the rule base. In addition to that, there are some parameter settings that must be modified in the MATLAB code of optimization techniques, where the population size (N) is set to be (20), and the maximum number of iterations (T) is set to be (10). All experiments are conducted separately ten times for every optimization technique, and the mean of each time is used as the metric of optimization algorithm performance.

**Table 1:** Membership functions and rules of FLC.

Error	PB	PM	PS	ZE	NS	NM	NB
ΔError							
PB	PB	PB	PM	PM	PS	PS	ZE
PM	PB	PM	PM	PS	PS	ZE	NS
PS	PM	PM	PS	PS	ZE	NS	NS
ZE	PM	PS	PS	ZE	NS	NS	NM
NS	PS	PS	ZE	NS	NS	NM	NM
NM	PS	ZE	NS	NS	NM	NM	NB
NB	ZE	NS	NS	NM	NM	NB	NB

### Simulation process using GWO algorithm

**Fig. 8:**
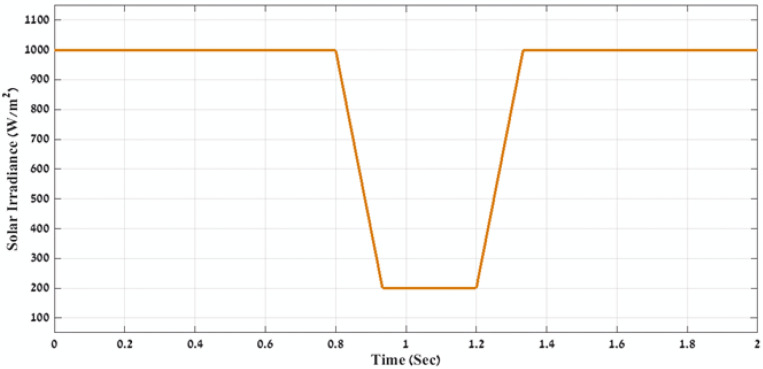
The applied solar irradiation levels on the simulated PV power plant.

**Fig. 9:**
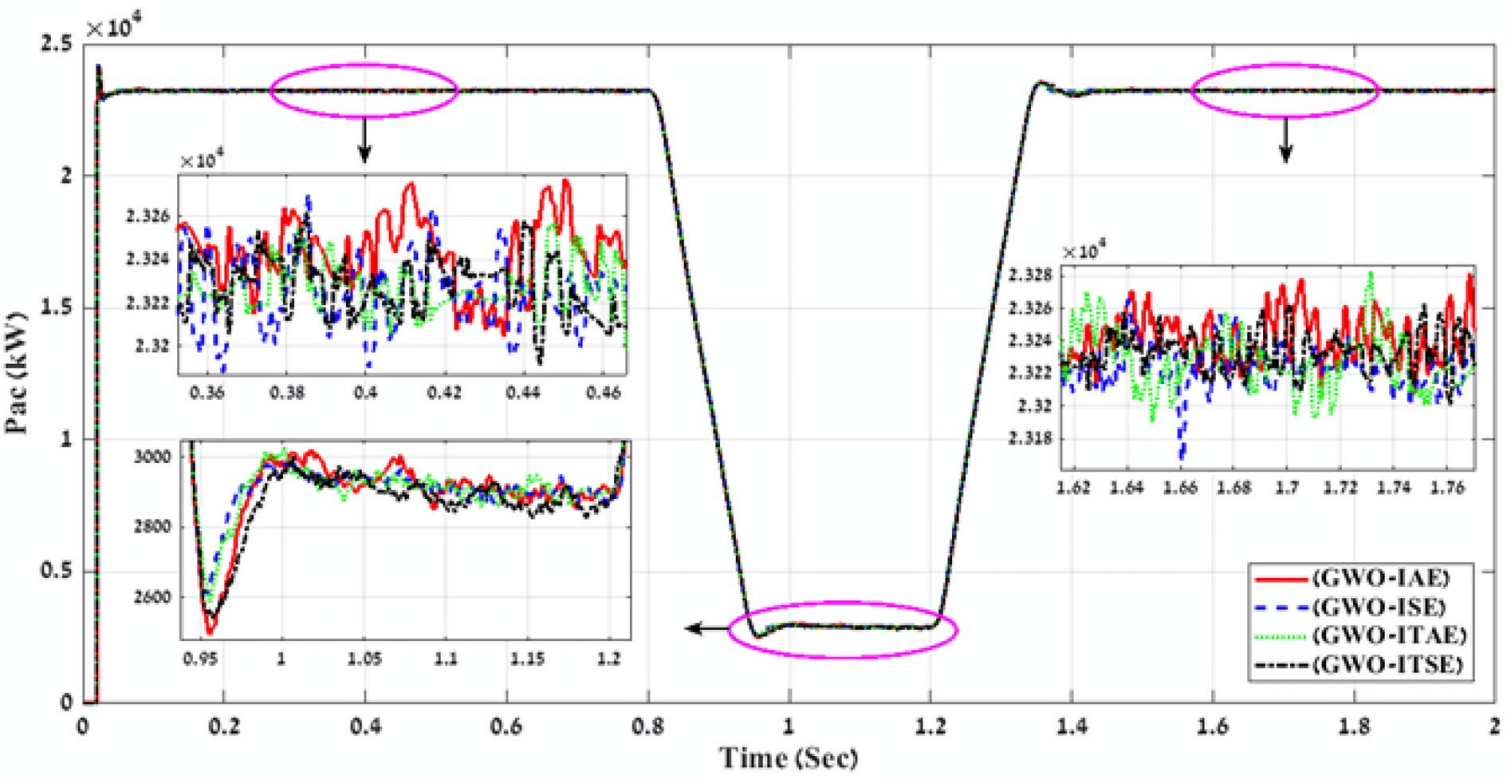
A comparison between the power curves of the simulated PV power plant in case of using the gain values obtained by GWO algorithm for FLC-PI control.

**Fig. 10:**
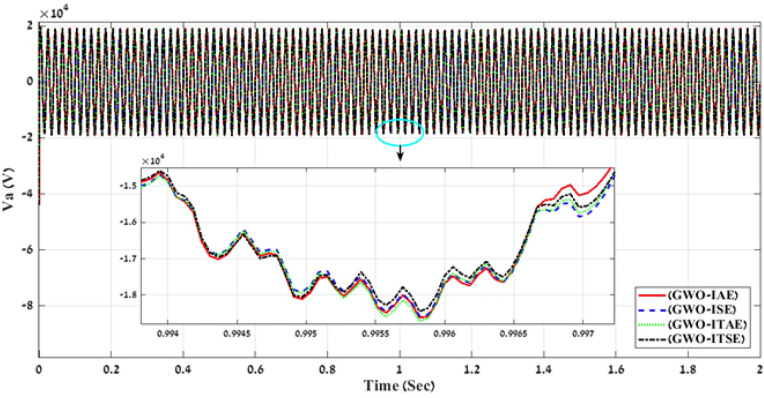
A comparison between the voltage curves of the simulated PV power plant in case of using the gain values obtained by GWO algorithm for FLC-PI control.

In the first stage, MATLAB code of GWO algorithm is applied through using the four-error benchmark objective functions (IAE, ISE, ITAE, and ITSE) on the simulated PV power plant during different solar irradiation levels as is shown in Fig. 8. Consequently, there are number of results that present the objective functions and gain values (K1, K2, K3, K4, K5, K6, K7, K8, K9, ki_VDCreg, kp_VDCreg, ki_Ireg, kp_Ireg) that can apply on the proposed FLC-PI control of this PV power plant. By applying all these gain values on the proposed FLC-PI control of this PV power plant during different solar irradiation levels, there is a best result obtained of each error as is shown in Table 2 . To define the best result of these gain values, there is a comparison between the generated voltage curves and power curves with time for running the simulated PV power plant during different solar irradiation levels in case of using each gain values of Table 2. According to THD values of the output voltage that are produced of applying gain values of Table 2 and Fig. 9 and Fig. 10, these comparisons approve that the best gain values that can apply on the proposed FLC-PI control of this PV power plant are obtained in case of using GWO algorithm with ITSE error as is marked in Table 2.

**Table 2:** Simulation results of the applied GWO algorithm in case of using Benchmark Errors (IAE, ISE, ITAE, and ITSE).

FLC-PI _GWO_(IAE, ISE, ITAE, ITSE)																			
Error type	Rise time	Setting time	Overshoot	Objective function	Ki_VDCreg	Kp_VDCreg	Ki_Ireg	Kp_Ireg	K1	K2	K3	K4	K5	K6	K7	K8	K9	THD %	Time taken
IAE	171.71	330.08	1.05	5276.16	273.00	1.64	19.59	0.31	0.60	0.27	1.05	0.58	0.91	0.86	0.76	1.19	0.62	4.01	216.04
ISE	141.28	330.08	0.54	272.37	465.89	3.03	20.34	0.24	0.99	1.15	0.77	0.57	0.53	0.53	1.29	0.42	0.79	3.98	246.50
ITAE	233.81	330.08	1.92	503.63	353.83	1.90	21.14	0.29	0.76	0.92	0.91	0.92	0.74	0.78	1.31	0.79	0.94	3.98	235.76
ITSE	228.87	330.09	1.52	21.66	210.20	2.60	21.72	0.30	0.89	0.94	0.97	0.71	0.54	0.70	1.38	0.15	0.68	3.97	248.60

### Simulation process using HHO algorithm

**Fig. 11:**
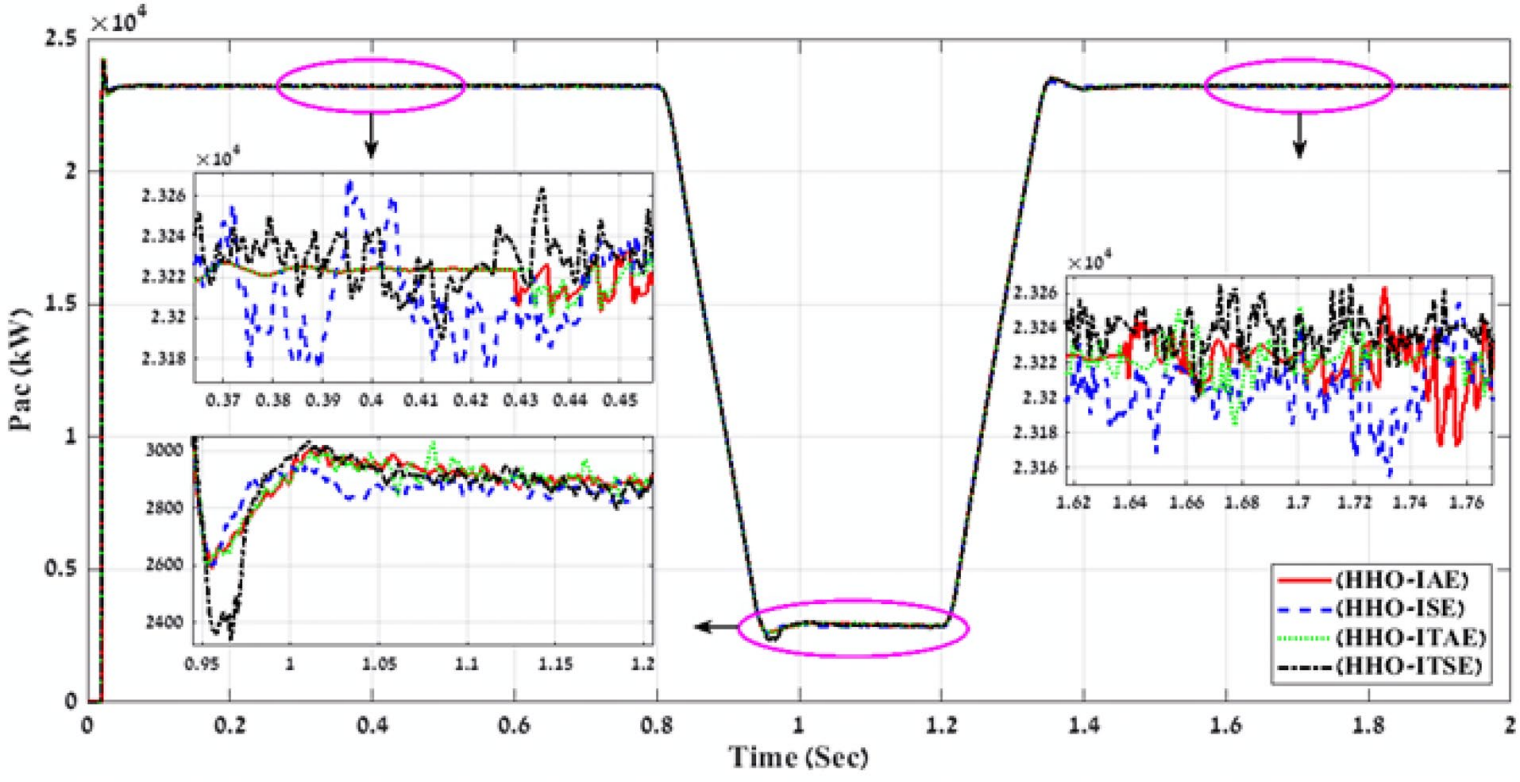
A comparison between the power curves of the simulated PV power plant in case of using the gain values obtained by HHO algorithm for FLC-PI control.

**Fig. 12:**
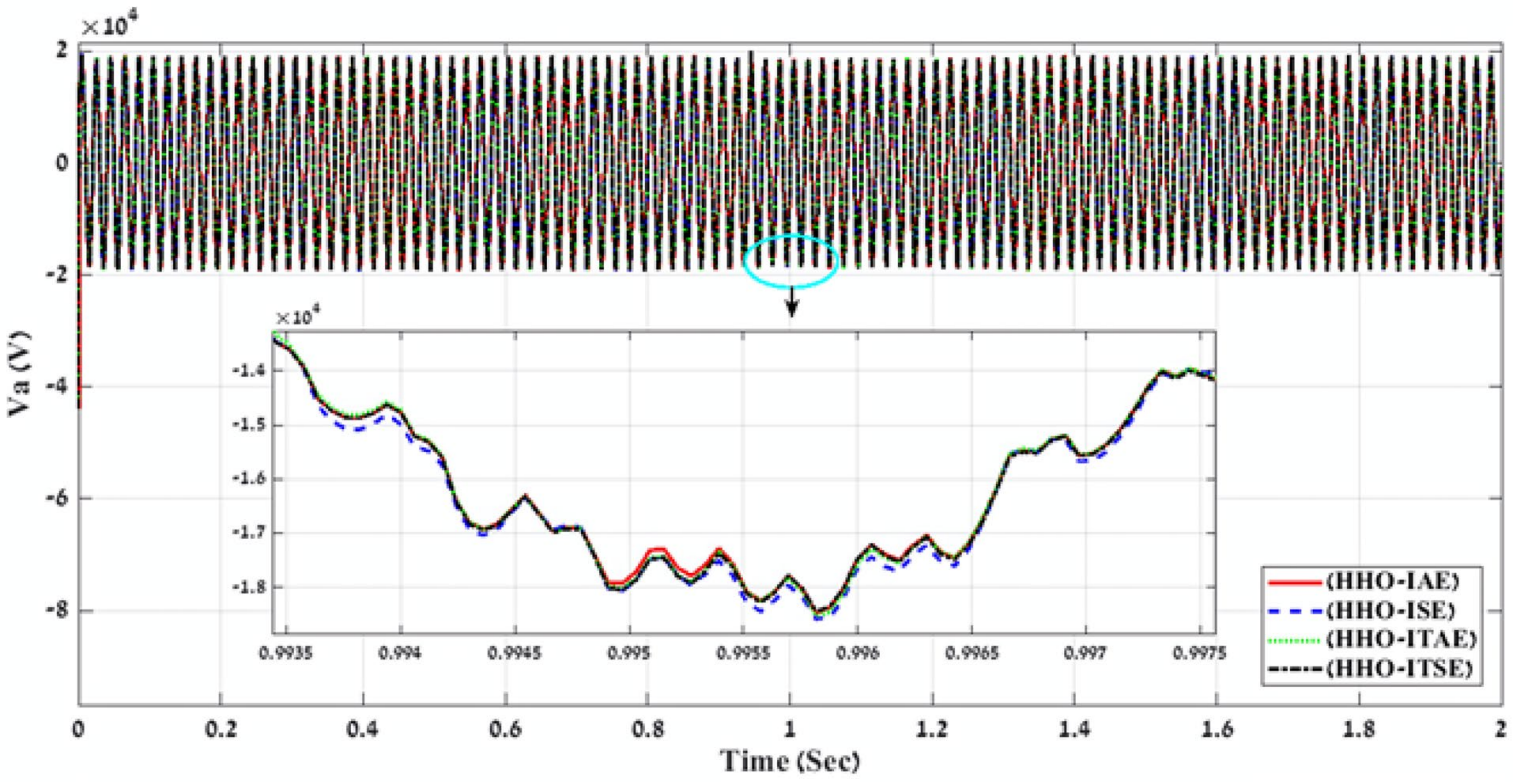
A comparison between the voltage curves of the simulated PV power plant in case of using the gain values obtained by HHO algorithm for FLC-PI control.

In the second stage, MATLAB code of HHO algorithm is applied through using the four-error benchmark objective functions (IAE, ISE, ITAE, and ITSE) on the simulated PV power plant during different solar irradiation levels as is shown in Fig. 8. Consequently, there are number of results that present the objective functions and gain values (K1, K2, K3, K4, K5, K6, K7, K8, K9, ki_VDCreg, kp_VDCreg, ki_Ireg, kp_Ireg) that can apply on the proposed FLC-PI control of this PV power plant. By applying all these gain values on the proposed FLC-PI control of this PV power plant during different solar irradiation levels, there is a best result obtained of each error as is shown in Table 3. To define the best result of these gain values, there is a comparison between the generated voltage curves and power curves with time for running the simulated PV power plant during different solar irradiation levels in case of using each gain value of Table 3. According to THD values of the output voltage that are produced of applying gain values of Table 3 and Fig. 11 and Fig. 12, these comparisons approve that the best gain values that can apply on the proposed FLC-PI control of this PV power plant are obtained in case of using HHO algorithm with ISE error as is marked in Table 3.

**Table 3:** Simulation results of the applied HHO algorithm in case of using benchmark errors (IAE, ISE, ITAE, and ITSE).

FLC-PI _HHO_(IAE, ISE, ITAE, ITSE)																			
Error type	Rise time	Setting time	Overshoot	Objective function	Ki_VDCreg	Kp_VDCreg	Ki_Ireg	Kp_Ireg	K1	K2	K3	K4	K5	K6	K7	K8	K9	THD %	Time taken
IAE	124.23	330.07	2.91	2438.15	248.26	2.95	19.08	0.24	0.62	0.59	0.53	0.74	0.39	0.58	0.78	0.27	0.79	4.00	260.83
ISE	105.79	1547.21	54.03	423.45	409.94	3.29	20.10	0.30	0.90	0.66	0.65	0.33	0.67	0.93	0.90	0.36	0.82	3.88	171.66
ITAE	124.23	330.07	2.91	568.97	248.26	2.95	19.08	0.24	0.62	0.59	0.53	0.74	0.39	0.58	0.78	0.27	0.79	4.00	266.51
ITSE	289.20	330.08	1.85	77.79	242.30	2.68	20.28	0.29	0.97	0.68	0.95	0.51	0.16	0.94	0.90	0.26	0.89	3.98	216.06

### Simulation process using AOA algorithm

**Fig. 13:**
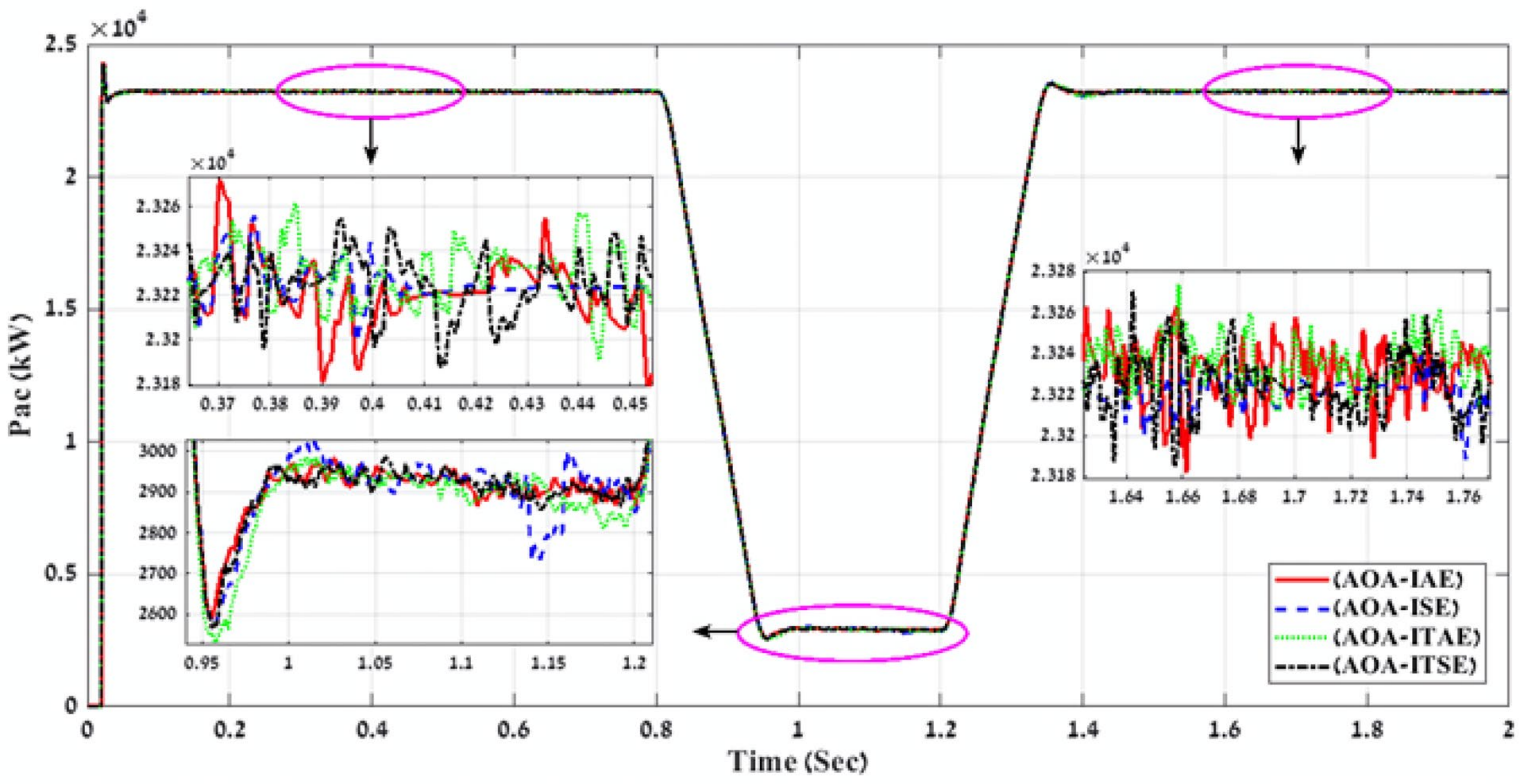
A comparison between the power curves of the simulated PV power plant in case of using the gain values obtain by AOA algorithm for FLC-PI control.

**Fig. 14:**
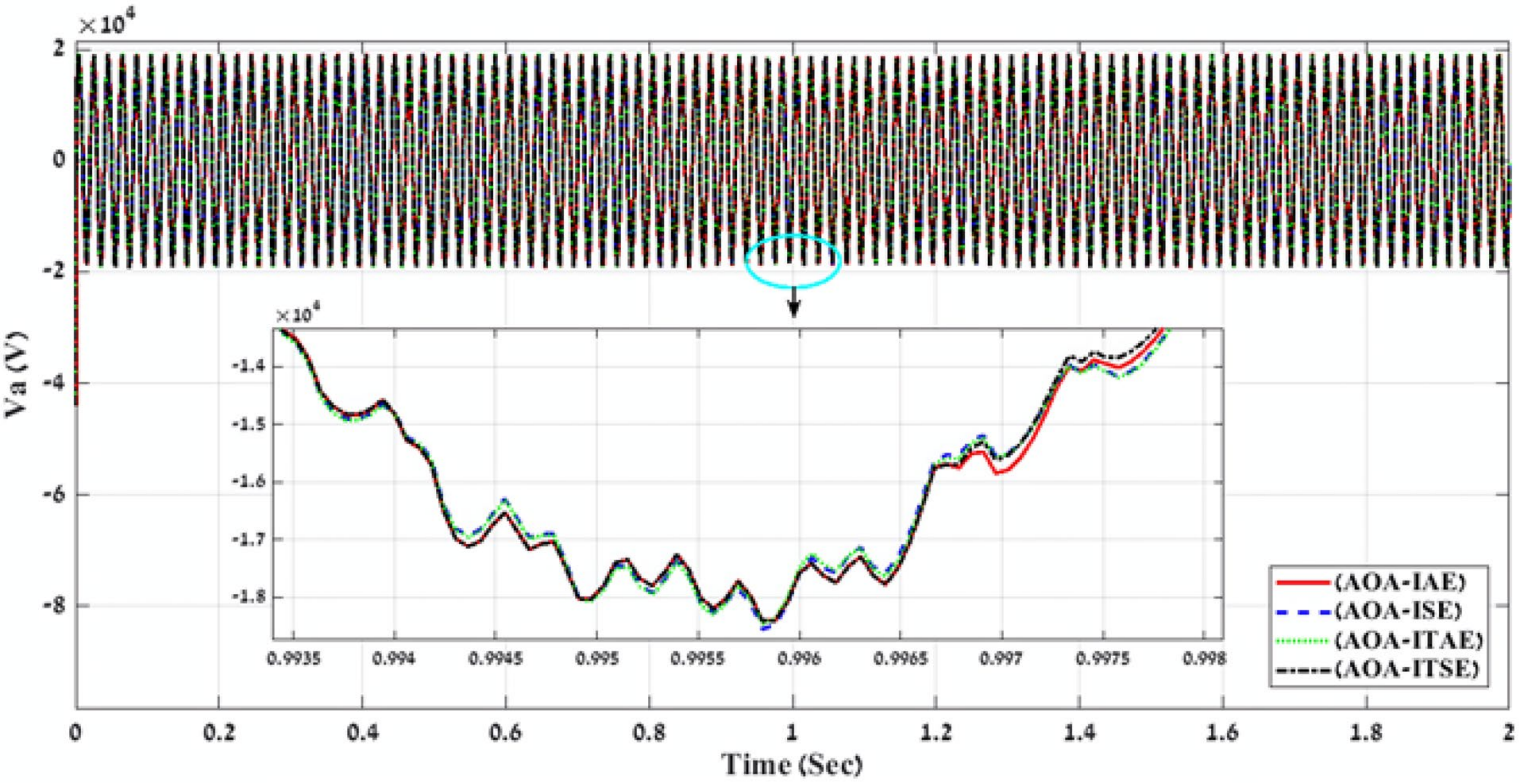
A comparison between the voltage curves of the simulated PV power plant in case of using the gain values obtained by AOA algorithm for FLC-PI control.

In the third stage, MATLAB code of AOA algorithm is applied through using the four-error benchmark objective functions (IAE, ISE, ITAE, and ITSE) on the simulated PV power plant during different solar irradiation levels as is shown in Fig. 8. Consequently, there are number of results that present the objective functions and gain values (K1, K2, K3, K4, K5, K6, K7, K8, K9, ki_VDCreg, kp_VDCreg, ki_Ireg, kp_Ireg) that can apply on the proposed FLC-PI control of this PV power plant. By applying all these gain values on the proposed FLC-PI control of this PV power plant during different solar irradiation levels, there is a best result obtained of each error as is shown in Table 4. To define the best result of these gain values, there is a comparison between the generated voltage curves and power curves with time for running the simulated PV power plant during different solar irradiation levels in case of using each gain value of Table 4. According to THD values of the output voltage that are produced by applying gain values of Table 4 and also Fig. 13 and Fig. 14, these comparisons approve that the best gain values that can apply on the proposed FLC-PI control of this PV power plant are obtained in case of using AOA algorithm with ITAE error as is marked in Table 4.

**Table 4:** simulation results of the applied AOA algorithm in case of using benchmark errors (IAE, ISE, ITAE, and ITSE).

FLC-PI _AOA_(IAE, ISE, ITAE, ITSE)																			
Error type	Rise time	Setting time	Overshoot	Objective function	Ki_VDCreg	Kp_VDCreg	Ki_Ireg	Kp_Ireg	K1	K2	K3	K4	K5	K6	K7	K8	K9	THD %	Time taken
IAE	133.13	330.07	2.75	2039.03	332.34	2.65	20.86	0.23	0.77	0.64	0.59	0.79	0.71	0.12	1.23	0.27	0.18	3.98	243.77
ISE	149.81	330.07	0.47	489.53	238.45	2.52	21.15	0.25	0.51	0.52	0.97	0.61	0.27	0.44	0.57	0.29	0.57	4.00	239.02
ITAE	228.87	330.09	1.52	549.90	210.20	2.60	21.72	0.30	0.89	0.94	0.97	0.71	0.54	0.70	1.38	0.15	0.68	3.97	249.70
ITSE	135.71	330.07	3.61	55.82	295.57	2.80	21.15	0.23	0.83	0.57	0.54	0.82	0.86	0.17	1.02	0.14	0.53	3.99	251.51

### Results analysis

**Fig. 15:**
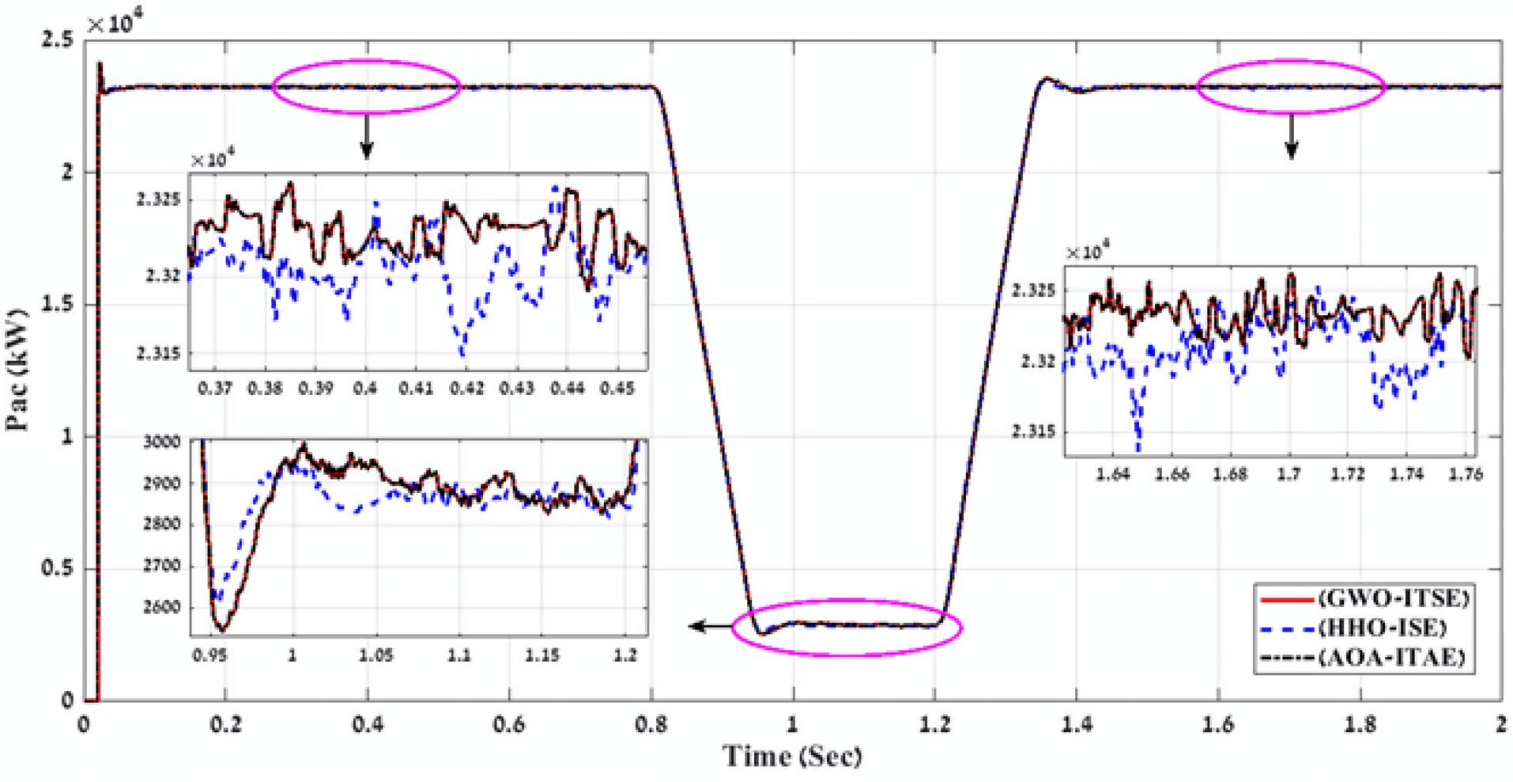
A comparison between the power curves of the simulated PV power plant in case of using the best gain values obtained by GWO, HHO and AOA algorithms for FLC-PI control.

**Fig. 16:**
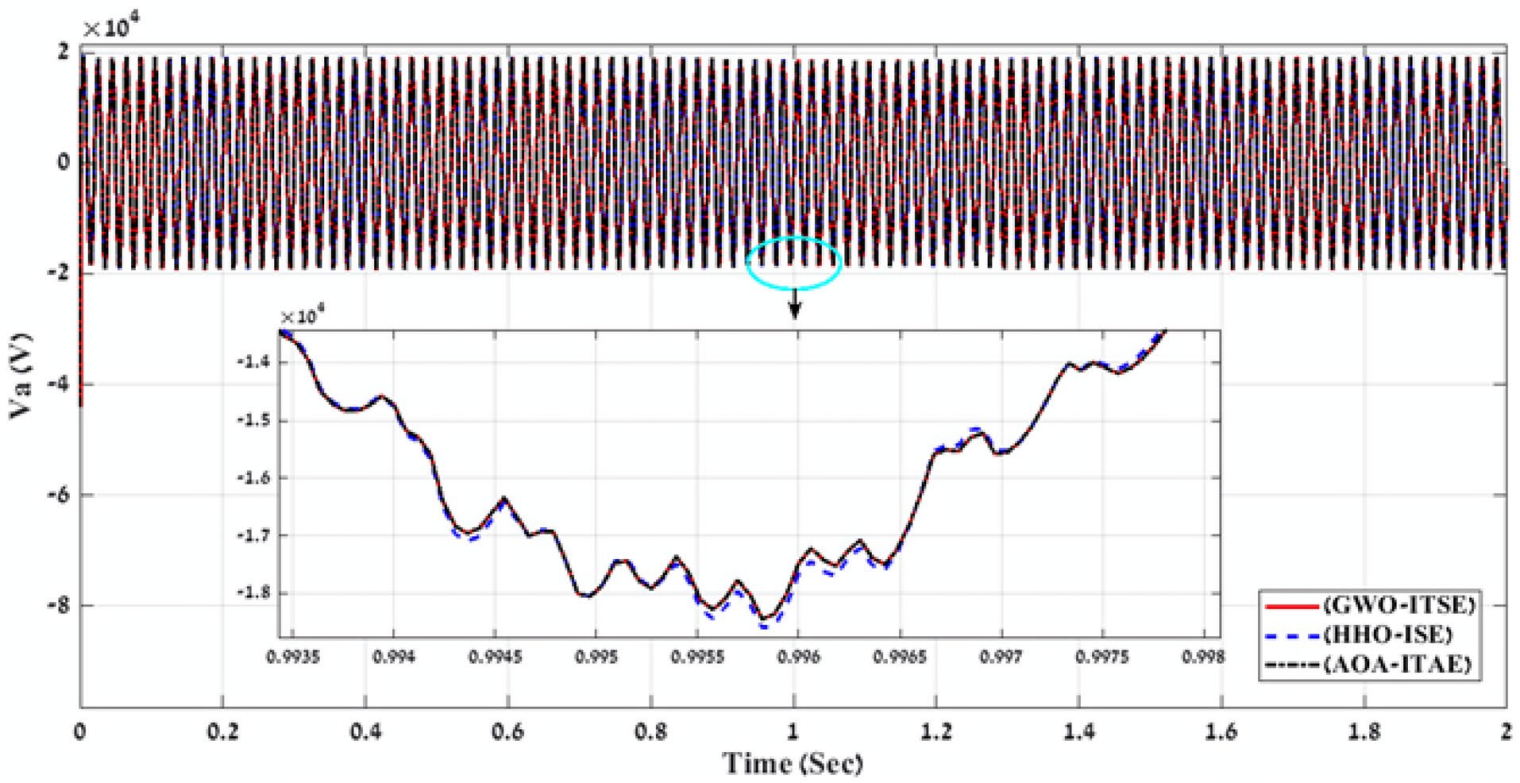
A comparison between the voltage curves of the simulated PV power plant in case of using the best gain values obtained by GWO, HHO and AOA algorithms for FLC-PI control.

To define the best optimization algorithm and error type that must be used together to tune and optimize all gain values of the proposed FLC-PI control for the simulated PV power plant then generating the best perfect gain values. All the gain values (K1, K2, K3, K4, K5, K6, K7, K8, K9, ki_VDCreg, kp_VDCreg, ki_Ireg, kp_Ireg) of Table 2, 3, 4 are applied on the proposed FLC-PI control of the simulated PV power plant during different solar irradiation levels. In addition, to do comparisons between the generated voltage curves and power curves with time for running the simulated PV power plant in case of using all gain values of Table 2, 3, 4. The best gain values that can apply on the proposed FLC-PI control of this PV power plant are defined as is shown in Table 5. Finally, according to the lower THD value of output voltage that is produced by running all gain values of Table 4. and Fig. 15 and Fig. 16. The best perfect gain values that must apply on the proposed FLC-PI control of this PV power plant during different solar irradiation conditions are obtained in case of using HHO algorithm with ISE error as marked in Table 5. To define the dynamic performance of the proposed FLC-PI control in case of tuning its gain values using MOTs. There is a comparison between applying this FLC-PI controller and PI controller on the simulated PV power plant during tuning these gain values through using the trial error and optimization methods as is shown in Table 6^69^. This comparison approves that applying the proposed FLC-PI control on the simulated PV power plant in case of tuning its gain value using MOTs is the best case where the high dynamic performance and the lower THD value of the output voltage are achieved as is marked in Table 6 . Also, in this work a comparison is made between all the produced THD percent at the output medium voltage (22 kV) of the simulated PV power plant as is shown in Table 6 and the international standard THD percent of (IEEE 519–2014) limits according to the medium voltage level in the electric power systems as is marked in Table 7^70^. This comparison shows that the most suitable case for reducing THD in the power system signals is achieved by applying HHO algorithm with ISE error to optimize gain values of the proposed FLC-PI control for the simulated PV power plant, where this case achieves THD percent equal 3.88% (less than the international standard limit for the medium voltage = 5.00%). Finally, all the results and comparisons of this paper are concluded that it is recommended to apply the proposed FLC-PI control in case of tuning its gain values using MOTs specially HHO algorithm with ISE error for enhancing the voltage stability, power quality and the overall performance of the high penetration of PV power plant into the utility grid, in addition to reduce the power losses especially with the fluctuated solar irradiation levels.

**Table 5:** Best gain values obtain by GWO, HHO and AOA algorithms using Benchmark errors for FLC-PI control.

MOTs—error type	Rise time	Setting time	Overshoot	Objective function	Ki_VDCreg	Kp_VDCreg	Ki_Ireg	Kp_Ireg	K1	K2	K3	K4	K5	K6	K7	K8	K9	THD %	Time taken
GWO -ITSE	228.87	330.09	1.52	21.66	210.20	2.60	21.72	0.30	0.89	0.94	0.97	0.71	0.54	0.70	1.38	0.15	0.68	3.97	248.60
HHO -ISE	105.79	1547.21	54.03	423.45	409.94	3.29	20.10	0.30	0.90	0.66	0.65	0.33	0.67	0.93	0.90	0.36	0.82	3.88	171.66
AOA -ITAE	228.87	330.09	1.52	549.90	210.20	2.60	21.72	0.30	0.89	0.94	0.97	0.71	0.54	0.70	1.38	0.15	0.68	3.97	249.70

**Table 6:** THD obtain of the simulated PV power plant after tuning gains of the used control with different methods.

Tuning method	Control type	THD (%)
*Trial and error*	PI	12.22
	Proposed FLC-PI	8.71
*Optimization*	PI (AOA-ISE)	7.25
	Proposed FLC-PI (HHO-ISE)	3.88

Note: High-voltage systems can have up to 2.0% THD where the cause is an HVDC terminal whose effects will have attenuated at points in the network where future users may be connected.

**Table 7:** International standard limits of voltage harmonic distortion in the electric power systems (IEEE 519–2014).

Bus voltage at point of common coupling (PCC)	Individual voltage distortion (%)	Total voltage distortion THD (%)
V ≤ 1.0 kV	5.0	8.0
1 kV < V ≤ 69 kV	3.0	5.0
69 kV < V ≤ 161 kV	1.5	2.5
161 kV < V	1.0	1.5

## Conclusion

This paper presents a detailed simulation study of a 26.136 MWp grid-connected photovoltaic power plant (PVPP), already integrated into the Egyptian national grid at Fares City, Kom Ombo Centre, Aswan Governorate. The PVPP consists of eleven identical blocks and a point of connection to the utility grid, modeled in MATLAB/SIMULINK using actual field data. Each block comprises 2376 kWp PV arrays interfaced with DC-DC boost converters, which regulate the output DC power. A central inverter converts the regulated DC power into AC in accordance with grid code requirements. A hybrid control method, combining Fuzzy Logic Control (FLC) with a Proportional-Integral (PI) controller, is proposed to enhance the dynamic performance of the central inverters. This approach aims to improve both voltage stability and power quality under varying solar irradiance conditions. To ensure optimal controller performance, several artificial intelligence (AI)-based metaheuristic optimization techniques (MOTs) are employed to simultaneously tune all control parameters, including *Kp*, *Ki* (for the PI controller) and *K*1 through *K*9 (for the FLC). Three MOTs—Grey Wolf Optimization (GWO), Harris Hawks Optimization (HHO), and Arithmetic Optimization Algorithm (AOA)—are used to identify the optimal FLC-PI control gains, leveraging four classical error-based objective functions: Integral Absolute Error (IAE), Integral Squared Error (ISE), Integral Time Absolute Error (ITAE), and Integral Time Squared Error (ITSE). The simulation methodology includes a comparative analysis between the conventional PI controller and the proposed FLC-PI control, with controller gains tuned through both trial-and-error and MOTs. Simulation results demonstrate that the FLC-PI controller optimized using MOTs significantly outperforms the conventional PI controller, delivering faster dynamic response, enhanced voltage stability, and reduced Total Harmonic Distortion (THD). Furthermore, a comparative analysis of the output voltage THD against the IEEE 519–2014 standard for medium voltage levels shows that the most effective configuration is the HHO algorithm optimized with the ISE objective function, achieving a THD of 3.88%, which is well below the 5.00% limit. Based on the simulation findings, it is strongly recommended to implement the proposed FLC-PI control for high-penetration PVPPs connected to the utility grid. The integration of MOTs—especially HHO with the ISE criterion—provides the best solution for enhancing the performance of central inverters under varying solar conditions. Additionally, proper tuning of the built-in LC filters in the central inverters is advised to mitigate voltage transients and power fluctuations, particularly under partial shading scenarios.

## Future work

Although the proposed FLC-PI control optimized by metaheuristic optimization techniques (GWO, HHO, and AOA) has shown promising results in enhancing the performance of the 26.136 MWp grid-connected PV power plant, several directions for future research can be pursued: (i) Hardware implementation and real-time validation for implementing the proposed FLC-PI control algorithm on actual hardware using real-time simulators (e.g., OPAL-RT, dSPACE) to validate the simulation results and ensure its effectiveness in practical grid-connected PV systems; (ii) Multi-objective optimization that simultaneously consider trade-offs between power quality metrics (e.g., THD, voltage deviation), operational cost, energy yield, and inverter efficiency; (iii) Hybrid metaheuristic approaches via hybridization of MOTs (e.g., combining HHO with PSO or AOA with GWO) to overcome the limitations of individual algorithms such as premature convergence or getting trapped in local optima, especially under complex operating conditions; (iv) Adaptive and self-tuning controllers, such as reinforcement learning or adaptive fuzzy-neural networks, could further enhance the robustness of the controller under various uncertainty and dynamic conditions.

## Data Availability

The authors would like to confirm that all data generated or analyzed during this study are included within the entire text of the presented article.
